# Molecular Characterization of *Philometra obladae* (Nematoda: Philometridae) in Juvenile *Oblada melanura* (Linnaeus, 1758) from the Tyrrhenian Sea off Sicily, Italy

**DOI:** 10.3390/pathogens13110971

**Published:** 2024-11-06

**Authors:** Giovanni De Benedetto, Kristian Riolo, Emanuela Sturiale, Alessia Giannetto, Gabriella Gaglio

**Affiliations:** 1 Department of Veterinary Sciences, University of Messina, 98168 Messina, Italy; gdebenedetto@unime.it (G.D.B.); esturiale@unime.it (E.S.); 2 Department of Chemical, Biological, Pharmaceutical and Environmental Sciences, University of Messina, 98166 Messina, Italy; kristian.riolo@unime.it (K.R.); agiannetto@unime.it (A.G.)

**Keywords:** Nematoda, *Philometra* spp., *small subunit 18S rRNA*, *cytochrome c oxidase 1 cox1*, saddled seabream

## Abstract

*Philometra obladae* is a nematode belonging to the family Philometridae. It was morphologically described for the first time in 2008 in *Oblada melanura*. To date, few data on the molecular characterization of Philometridae have been reported. The aim of the present study was to molecularly characterize *Philometra obladae* in *O. melanura* inhabiting the Tyrrhenian coasts off Sicily, Italy. In July 2023, five nematodes were found and morphologically identified as *Ph. obladae* from the celomic cavity of four *O. melanura* specimens. Genomic DNA from four nematodes was extracted and two molecular markers, the ribosomal *18S rRNA* and the mitochondrial *cox1*, were amplified using polymerase chain reaction. The sequences obtained were aligned using the MUSCLE algorithm and were used for phylogenetic analyses. Partial sequences of both markers were submitted to GenBank. Phylogenetic trees for both markers resulted in very similar topologies with high posterior probabilities and bootstrap values. Comparisons of our results indicated that *Ph. obladae* is related to the sequences of other Philometridae isolated from different hosts and different geographic areas. Phylogenetic analysis was carried out to compare the sequences of *Ph. obladae* with other marine Philometridae, which allowed for the molecular characterization of *Ph. obladae* as an independent species for the first time.

## 1. Introduction

Since 1960, the genus *Philometra* Costa, 1845 has included over 100 species reported in several freshwater, brackish and seawater teleost hosts worldwide. This genus is considered the largest group in the family Philometridae [1,2,3,4,5]. Dracunculoid nematodes belonging to the genus *Philometra* have been particularly poorly investigated so far. Thus, data on their species grouping is currently considered insufficient and need updating and expansion [2,6]. While the morphological description of many females and larvae of *Philometra* spp. has been reported, little is known regarding the males of these species. The *Philometra* sp. male is only seasonally present, making evaluation of the morphological and molecular characteristics complicated [2,7]. Morphological evaluation has been reported in only a few cases, and was related to the sampling period, e.g., eight *Philometra filiformis* Stossich, 1896 male specimens found in the ovary of a common pandora, *Pagellus erythrinus* Linnaeus, 1758 from the Tyrrhenian coast off Sicily, Italy, morphologically described by Gaglio and colleagues in June 2009 [8]. Some studies on *Philometra* sp. infection have been carried out in the Mediterranean basin. Among them, one *Ph. filiformis* gravid female specimen was described by Moravec and colleagues [9] in the ovary of *P. erythrinus* sampled from the central Mediterranean Sea. During the same survey, one specimen, morphologically identified as *Philometra obladae* n. sp. Moravec, Gaglio, Panebianco, Giannetto, 2008, was found in a *Oblada melanura* Linnaeus, 1758 celomic cavity. Another report of *Philometra spicarae* sp. n. was made by Moravec and colleagues [10] in the celomic cavity of picarel (*Spicara smaris*; Linnaeus, 1758) caught in the Ionian Sea (Sicily, Italy). Since 2005, studies including morphological and molecular information have provided evidence for a philometrid host specificity, showing the necessity to reevaluate the classification of nematodes belonging to this family [3,11,12]. In general, mainly for philometrid nematodes, integrative taxonomy may not be sufficient to evaluate biodiversity and provide differential characteristics to explain the taxonomic/phylogenetic differences between different species belonging to the same group [13,14]. Additional criteria that would be useful for providing information on nematode species differences must also include habitat variety, physiological markers, and/or ecological and population parameters [14]. Molecular data on nematodes belonging to the genus *Philometra* have been reported for a limited number of species [13,14,15,16]. Among them, sequences of cytochrome c oxidase 1 (*cox1*), previously used for this evaluation in other organisms [17], were applied in identifying philometrid nematodes [14], considering this gene to be the first choice for species differentiation [13]. Some authors have provided new molecular data on *Philometra* spp. using the previously reported *cox1* marker, which is associated with the small nuclear ribosomal subunit (*18S rRNA*); both markers allow for a better description of these parasites molecularly, resolving any taxonomic/phylogenetic discrepancies between sister species belonging to the same group [3,12,13,14,18]. According to the data reported above, mainly considering the necessity to associate morphological and molecular analyses to better describe parasite species and improve current knowledge, the aim of the present study is to associate molecular description with the previously morphologically described nematodes belonging to the species *Ph. obladae* [9,19] found in *O. melanura* juvenile specimens caught from the Tyrrhenian Sea, Sicily (Italy), using markers that are considered the gold standard for this purpose.

## 2. Materials and Methods

### 2.1. Fish Sampling and Parasitological Analysis

In July and August 2023, four *Oblada melanura* were found dead on the seabed during a dive off the coast of Capo d’Orlando, Messina Province, Tyrrhenian Sea (38°09′25.1″ N, 14°45′38.0″ E). At the same time, other specimens showed some abnormalities in their swimming patterns associated with abnormal celomic cavity distension. Sampled fish were processed via the opening of the celomic cavity and examined for *Philometra* sp. nematode presence according to Arthur and Albert [20]. The coelomic cavity and organs of each specimen were observed both macroscopically and with the aid of a stereomicroscope (Stereo Discovery.V12 Zeiss, Jena, Germany) using Petri dishes. The parasites were washed three times in saline solution and fixed in 70% ethanol for morphological evaluation following the key suggested by Moravec and colleagues [9]. Samples used for molecular analysis were stored at −80 °C using the protocol suggested by de Buron et al. [14] and Wang and colleagues [13], appropriately modified according to the requirements.

### 2.2. DNA Isolation and Polymerase Chain Reaction

Genomic DNA from four parasite specimens was extracted and purified using the DNeasy^®^ Blood and Tissue Kit (QIAGEN, Hilden, Germany), according to the manufacturer’s protocol. The genomic DNA obtained was processed to evaluate 2 different markers, namely the ribosomal DNA marker *18S ribosomal RNA* (*18S rRNA*) and the mitochondrial *cytochrome c oxidase 1* (*cox1*) gene, using polymerase chain reaction (PCR). The loci of interest were amplified using the primer sets listed in Table 1 and the recombinant Taq DNA polymerase (FIREPol^®^ DNA Polymerase Kit, Teaduspargi, Estonia) according to the manufacturer’s instructions. For each sample, a 50 μL reaction mixture containing 0.035 U/μL FIREPol^®^ DNA Polymerase, 1x FIREPol^®^ Buffer BD, 200 μM of dNTP mix, 2 mM MgCl_2_, and 0.2 μM primers (forward and reverse) was prepared. PCR reactions were performed in an Ep-Gradient Mastercycler (Eppendorf, Hamburg, Germany) using the following cycling parameters for *18S rRNA*: 95 °C for 5 min, 34 cycles of 95 °C for 30 s, 54 °C for 1 min, and 72 °C for 2 min, followed by one final step of 55° for 2 min and 72 °C for 8 min. For *cox1* the following profile was performed: 95 °C for 4 min, 2 cycles of 95 °C for 30 s, 45 °C for 30 s, 72 °C for 30 s, followed by 40 cycles of 95 °C for 30 s, 51 °C for 1 min and 72 °C for 1 min, final extension was performed at 72 °C for 10 min. PCR products (expected sizes 900 and 400 bp for *18S rRNA* and *cox1*, respectively) were analysed via 1.5% agarose gel electrophoresis and the successfully amplified samples were then purified using the E.Z.N.A. gel extraction kit (Omega Bio-tek, Norcross, GA, USA). DNA quantity and purity were verified by UV absorbance measurements at 260, 230, and 280 nm (NanoDrop 2000, Thermo Scientific, Wilmington, MA, USA).

### 2.3. DNA Sequencing and Alignment

The DNA amplicons obtained were sequenced in both the forward and reverse directions using an Applied Biosystems 3730 DNA analyser (Thermo Fisher Scientific, Waltham, MA, USA), and the DNA sequences obtained were analysed by BLASTN similarity search compared to the NCBI database (http://blast.ncbi.nlm.nih.gov/Blast.cgi, accessed on 8 June 2024).

The *18S* rRNA and *cox1* sequences obtained from the isolates were aligned with the available nucleotide sequences of Philometridae (Table 2) using the MUSCLE algorithm and further used for phylogenetic analyses. Neighbour-joining (NJ), maximum likelihood (ML), and Bayesian inference (BI) trees were constructed by selecting the GTR + G + I nucleotide replacement model for all datasets, using the bootstrap method (1000 replications) to evaluate the consistency of internal divisions, following the model selection described by Lefort et al. [22]. NJ and ML phylogenetic analyses were performed using MEGA X and PhyML 3.0, respectively [23,24]; BI analysis was carried out using MrBayes 3.2.6 [25] with the GTR model with the same rates (1,000,000 generations, sampling every 500 generations and burn-in fraction 0.25). The obtained trees were visualized using the iTOL online tool [26] and fixed with *Dracunculus insignis* Leidy, 1858 (*18S rRNA*) and *Philometra carolinensis* Moravec, de Buron, Roumillat, 2006 (*cox1*) chosen as outgroups.

## 3. Results

The celomic cavities of the investigated *O. melanura* juvenile specimens showed the presence of five adult nematodes. Collected specimens’ mean length was 186 ± 16 mm; cephalic and caudal mean widths were 1.17 ± 0.4 mm and 1.14 ± 0.2 mm, respectively. Morphological measurements and features of all examined specimens were compared with the previous description [9], allowing for the identification of all parasites as *Philometra obladae* mature females, as reported by De Benedetto and Gaglio [19]. Molecular and phylogenetic results are reported below.

### Molecular Analysis

**Figure 1:**
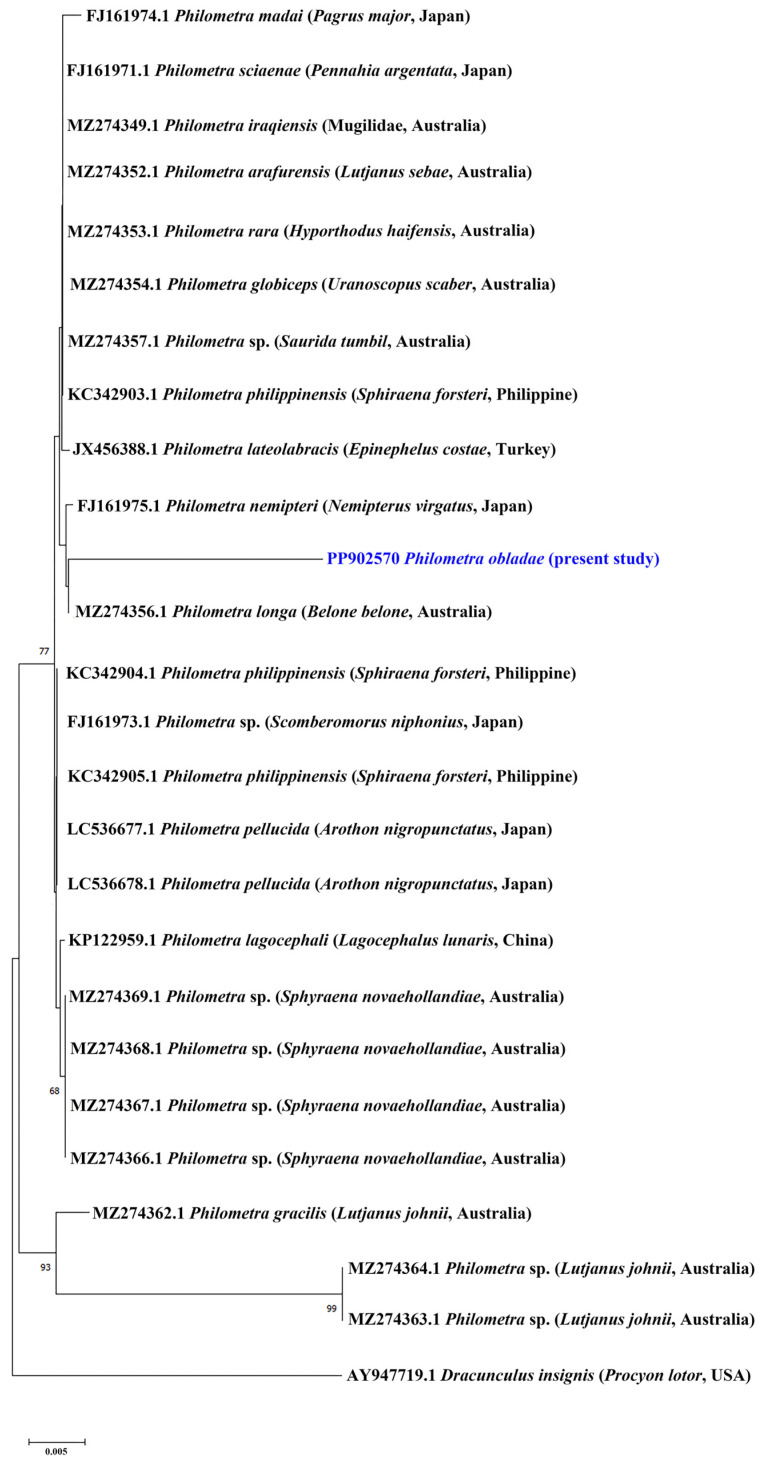
Phylogenetic relationships among the isolates of the present study and other *Philometra* sp. as inferred from sequences of *18S rRNA* analysed via the ML method. Numbers at the nodes refer to ML analysis; only bootstrap values above 60% are shown. GenBank accession numbers are indicated before species names. The newly analysed species in this study is marked in blue. Isolation source and location information are reported in brackets. Outgroup: *Dracunculus insignis*.

**Figure 2:**
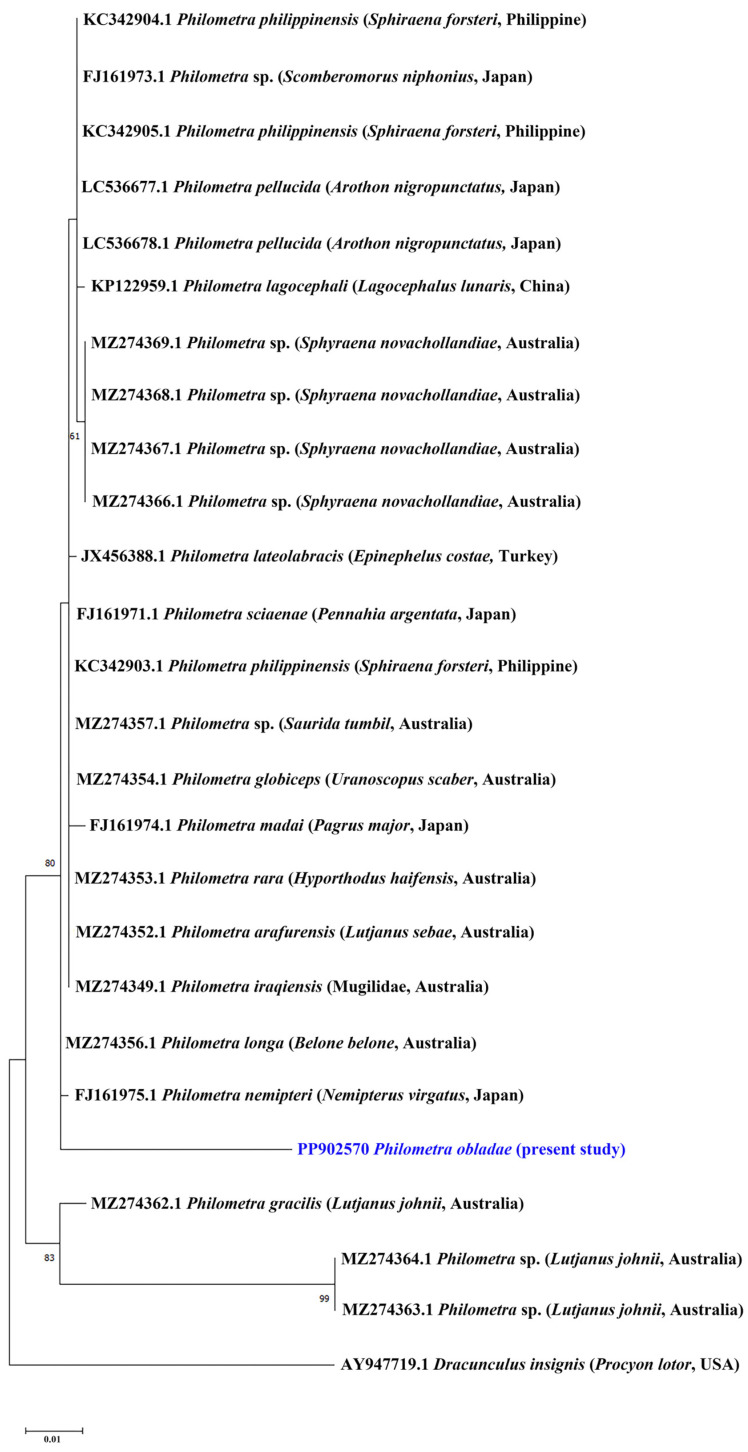
Phylogenetic relationships among the isolates of the present study and other *Philometra* sp. as inferred from the sequences of *18S rRNA* analysed via NJ. Numbers at the nodes refer to NJ analysis; only bootstrap values above 60% are shown. GenBank accession numbers are indicated before species names. The newly analysed species in this study is marked in blue. Isolation source and location information are reported in brackets. Outgroup: *Dracunculus insignis*.

**Figure 3:**
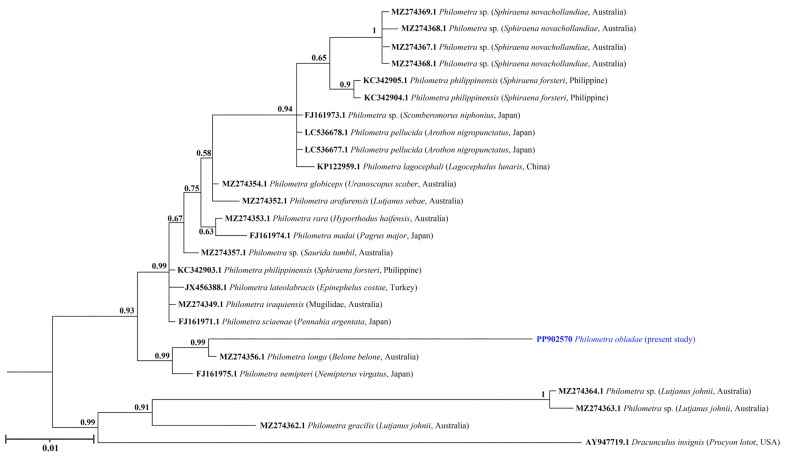
Phylogenetic relationships among the isolates of the present study and other *Philometra* sp. as inferred from sequences of *18S rRNA* analysed via the BI method. Numbers at the nodes (posterior probability values) refer to BI analysis. GenBank accession numbers are indicated before species names. The newly analysed species in this study is marked in blue. Isolation source and location information are reported in brackets. Outgroup: *Dracunculus insignis*.

**Figure 4:**
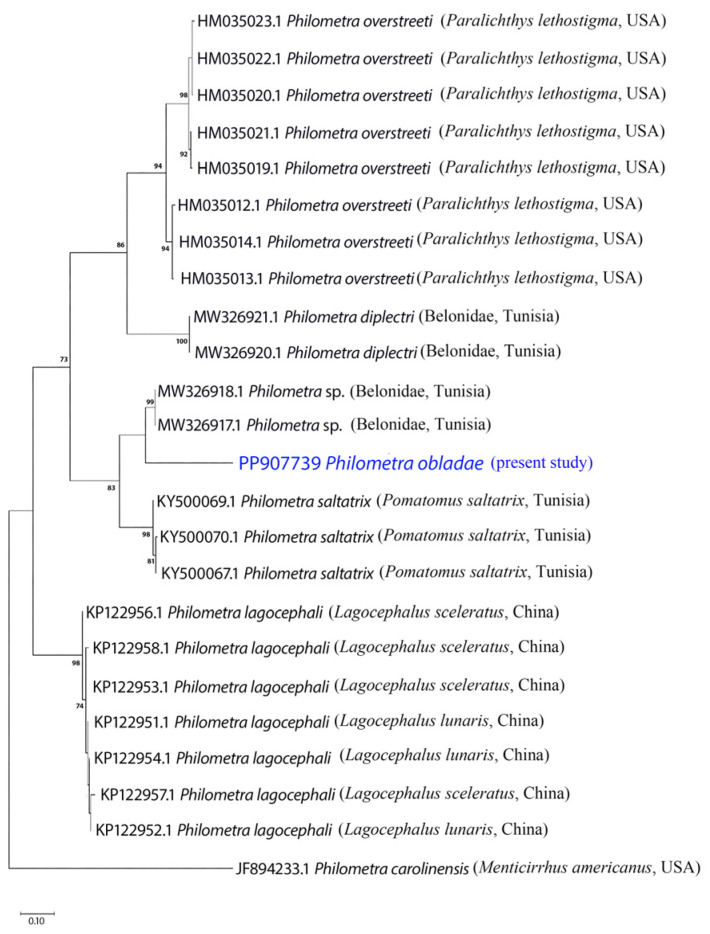
Phylogenetic relationships among the isolates of the present study and other *Philometra* sp. as inferred from sequences of *cox1* analysed via the ML method. Numbers at the nodes refer to ML analysis; only bootstrap values above 60% are shown. GenBank accession numbers are indicated before species names. The newly analysed species in this study is marked in blue. Isolation source and location information are reported in brackets. Outgroup: *Philometra carolinensis*.

**Figure 5:**
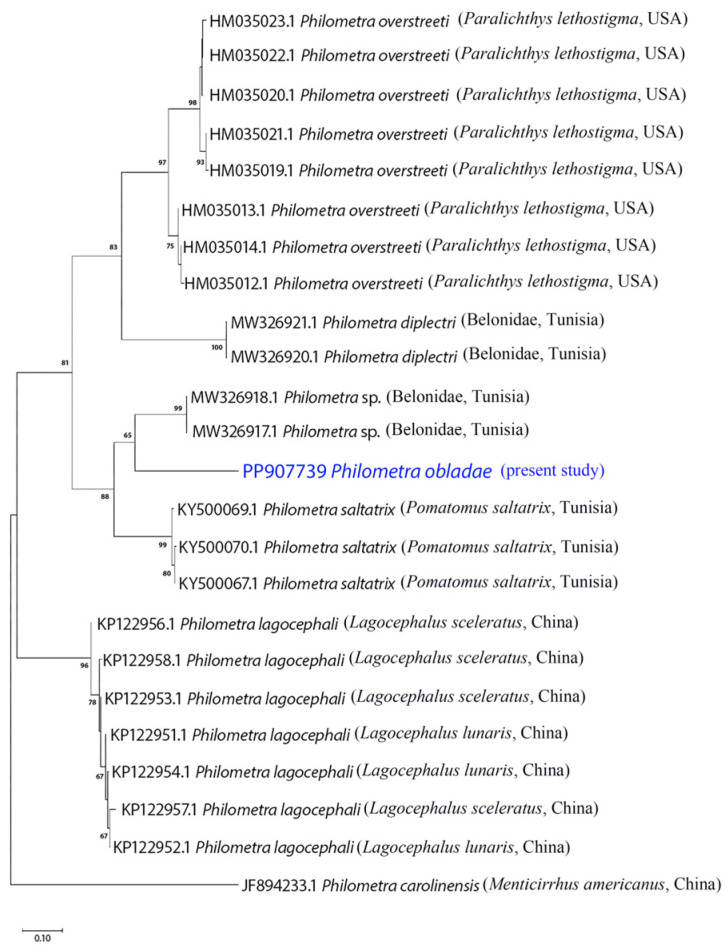
Phylogenetic relationships among the isolates of the present study and other *Philometra* sp. as inferred from sequences of *cox1* analysed via NJ. Numbers at the nodes refer to NJ analysis; only bootstrap values above 60% are shown. GenBank accession numbers are indicated before species names. The newly analysed species in this study is marked in blue. Isolation source and location information are reported in brackets. Outgroup: *Philometra carolinensis*.

**Figure 6:**
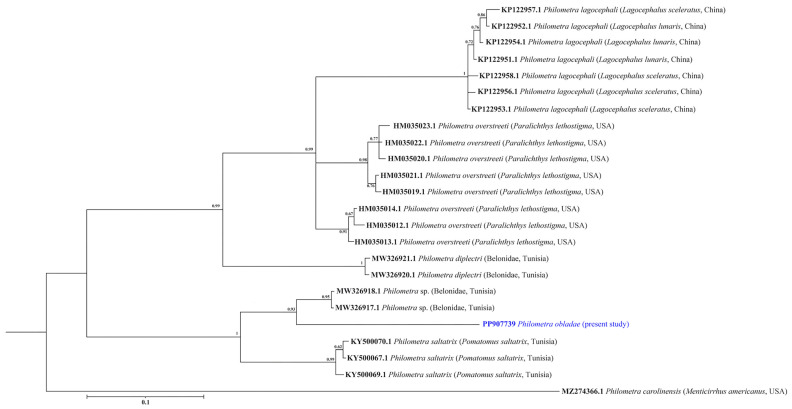
Phylogenetic relationships among the isolates of the present study and other *Philometra* sp. as inferred from sequences of *cox1* analysed via the BI method. Numbers at the nodes (posterior probability values) refer to BI analysis. GenBank accession numbers are indicated before species names. The newly analysed species in this study is marked in blue. Isolation source and location information are reported in brackets. Outgroup: *Philometra carolinensis*.

All isolates showed positive amplification for *18S* and *cox1* genes. Partial sequences of *18S rRNA* (823 nt, 5 replicates) and *cox1* (309 nt, 5 replicates) were obtained for *Ph. obladae*. The nucleotide sequences of the amplified products of each gene were identical among the isolates from all specimens collected. The representative DNA sequences for *18S rRNA* and *cox1* were submitted to GenBank (accession numbers PP902570 and PP907739, respectively). The representative sequences of *18S rRNA* had 94.45% and 93.73% similarity to that of *Philometra longa* Moravec, Barton and Shamsi, 2021 (MZ274356.1) and *Philometra* sp. (MW328560.1) from GenBank, with 49 and 55 nt differences, respectively. The obtained sequences of *cox1* had 84.67% similarity to *Philometra* sp. (OP221143.1) from GenBank, with 48 nt difference. The alignments of *18S rRNA* and *cox1* sequences used 667 and 242 informative positions for NJ, ML, and BI analyses. The dataset included sequences of representative seawater *Philometra* species deposited in GenBank. The phylogenetic trees for both *18S rRNA* and *cox1* markers resulted in a very similar topology, with reasonably high posterior probabilities and bootstrap values in most of the nodes. *Philometra obladae* (Philometridae) *18S rRNA* sequences grouped with *Philometra longa* (MZ274356.1) and *Philometra nemipteri* Luo, 2001 (FJ161975.1) (Figure 1, Figure 2 and Figure 3), while *Ph. obladae cox1* sequences grouped with *Philometra* sp. (MW326917.1) and *Philometra* sp. (MW326918.1). Comparisons of the results of two loci indicated that the sequences of *Ph. obladae* are related to those of other Philometridae isolated from different hosts and different geographic areas, such as *Ph. longa* and *Philometra* sp. from Belonidae, caught off Australia and the United States, respectively [6,29], and *Ph. nemipteri* from *Nemipterus virgatus* Houttuyn, 1782, caught off Japan [12] (Figure 4, Figure 5 and Figure 6).

## 4. Discussion

The present study provides the first molecular characterization of *Ph. obladae* in juvenile *O. melanura* specimens caught in the Tyrrhenian Sea off the Sicilian coast. The parasite was previously morphologically described by Moravec et al. [9]. The choice to investigate this parasite from the genetic point of view comes from a recent report provided by De Benedetto and Gaglio [19], who recorded more than 50% of saddled seabream showing an abnormal swimming behaviour associated with celomic swelling. These symptoms were attributable to parasitic infections and should be considered impactful to the *O. melanura* specimens present in the investigated area. As reported by Hanzelová and Moravec [2], a deep reassessment of the taxonomy and classification of dracunculoid parasites is necessary, combining molecular techniques with morphological examinations. The present study adds new data on the mitochondrial cytochrome c-oxidase 1 (*cox1*), associated with the small ribosomal subunit (*18S*) genes, to limit possible discrepancies in the phylogenetic analysis. In our study, *Ph. obladae* was characterized as an independent species, adding further information to its morphological description [9]. Molecular analysis involving the amplification of the *cox1* gene was carried out in accordance with de Buron and colleagues [14], with effective modifications to the thermal profile to obtain high-quality amplicons, which guaranteed a consistent sequencing process.

Regarding the trees constructed with the *18S rRNA* marker, the two methods used—ML and NJ—showed identical results. Specifically, the greatest homology with a bootstrap value exceeding 60 was observed with *Ph. longa* and *Ph. nemipteri*. The sequence of *Ph. longa* was reported by Barton and colleagues [28] in *Belone belone* Linnaeus, 1761 as the definitive host. The sequence of *Ph. nemipteri* was reported by Quiazon and collaborators [12] in *Nemipterus virgatus*. Both sequences refer to species with different geographical localizations: Australia and Japan for *Ph. longa* and *Ph. nemipteri*, respectively. Moreover, the definitive hosts of *Ph. longa* exhibit different feeding behaviours along the water column, supporting the hypothesis that there are different intermediate hosts for the various species of *Philometra* sp. However, the only description of the Philometridae life cycle has been reported by Bryan and colleagues [31], which identified *Oithona colcarva* Bowman, 1975, as the intermediate host of *Philometra overstreeti* Moravec and de Buron, 2006; therefore, the intermediate host of *Ph. obladae* remains unknown to date. The dietary patterns of *B. belone* are still poorly investigated. It is known to be carnivorous, and its diet varies according to geographical position and seasonality. Along the Eastern Adriatic coasts, the most common prey of *B. belone* are copepods [32,33], while in Irish waters, crab larvae and predominantly juvenile clupeids [33] make up its diet. In the Black Sea, *B. belone* feed on clupeids and anchovies [32,34,35]. Other components of its diet include molluscs, crustaceans, and isopods [36]. Unfortunately, also considering that *N. virgatus* is included in the IUCN Red List as Vulnerable [37], no data have been reported on *N. virgatus* ecology.

PCR for the *cox1* gene was carried out with significant adjustments to the first protocol reported by de Buron and colleagues [14], since the thermal profile was not useful for the amplification of our samples. *Philometra obladae cox1* sequences, compared with the previously reported sequences, allowed for the differentiation of our parasite well from the others. In particular, BLAST analysis highlighted the low percentage of identity (84.67%) between *Ph. obladae* and *Philometra* sp. found in teleosts belonging to the family Belonidae, confirming the high differentiation already reported for Dracunculoid parasites [2]. Moreover, the phylogenetic analysis carried out, comparing *Ph. obladae* to other seawater parasites belonging to the genus *Philometra*, allowed for the clear differentiation of our sequences from the others, highlighting proximity with two *Philometra* sp., also in this case, isolated from teleosts belonging to the family Belonidae; hosts showed net differences regarding geographical localization and feeding behavior, as reported by Kaya and Saglam [36]. According to Wang et al. [13], the mitochondrial marker *cox1* represents the gold standard for the phylogenetic characterization of nematode species belonging to the genus *Philometra*. According to the sequencing of our amplicons, we can confirm that the species characterization reported in the present study is fully superimposable on the previous reports. This finding is well supported by bootstrap values that made our analyses robust. Furthermore, although the *18S rRNA* marker is not reported as the first choice for species characterization, comparing our sequences with other studies [14,15,16], we can state that *18S* should be considered like *cox1* in terms of reliability of phylogenetic analysis and usefulness for species characterization.

## 5. Conclusions

The phylogenetic analysis carried out, comparing the sequences of *Ph. obladae* with other marine parasites belonging to the same genus, allowed us to molecularly differentiate this impactful parasite from *O. melanura* juveniles inhabiting the Tyrrhenian coasts off Sicily. Moreover, the definitive hosts of the *Philometra* species identified as belonging to *Ph. obladae* are characterized by dietary patterns that are highly differentiated from those of *O. melanura*, highlighting the role of different intermediate hosts, such as copepods, which should be investigated for all philometrid parasites. Furthermore, the possibility of designing a specific marker to be used for species characterization should be considered to allow for the rapid and complete identification of wild fish parasites.

## Figures and Tables

**Table 1:** List of targeted loci, sequence of the oligonucleotide forward (For) and reverse (Rev) primers used for PCR.

Locus	Sequence (5′–3′)
*18S rRNA* Floyd et al. [21]	For: CGCGAATRGCTCATTACAACAGC
	Rev: GGGCGGTATCTGATCGCC
*cox1* de Buron et al. [14]	For: CATTTRTTTTGRTTTTTTGG
	Rev: ACYACATRATAAGTATCRTG

**Table 2:** Nucleotide sequences of the *18S rRNA* and *cox1* markers used to evaluate the phylogenetic relations among our isolates and other Philometridae from seawater teleost.

Species	Isolate	Host	Locality	*18S*	*cox1*	Author
*Philometra saltatrix*	Posa6F1	*Pomatomus saltatrix*	Tunisia		KY500070.1	Moravec et al. [18]
*Philometra saltatrix*	Posa5F2	*Pomatomus saltatrix*	Tunisia		KY500069.1	Moravec et al. [18]
*Philometra saltatrix*	Muce6F1	*Pomatomus saltatrix*	Tunisia		KY500067.1	Moravec et al. [18]
*Philometra pellucida*	an1	*Arothron nigropunctatus*	Japan	LC536678.1		Iwaki et al. [27]
*Philometra pellucida*	am1	*Arothron nigropunctatus*	Japan	LC536677.1		Iwaki et al. [27]
*Philometra* sp.	SS-2020 Hapl2	Belonidae	USA		MW326918.1	Moravec et al. [6]
*Philometra* sp.	SS-2020	Belonidae	USA		MW326917.1	Moravec et al. [6]
*Philometra overstreeti*	W71	*Paralichthys lethostigma*	USA		HM035023.1	de Buron et al. [14]
*Philometra overstreeti*	W34	*Paralichthys lethostigma*	USA		HM035022.1	de Buron et al. [14]
*Philometra overstreeti*	W63	*Paralichthys lethostigma*	USA		HM035021.1	de Buron et al. [14]
*Philometra overstreeti*	W52	*Paralichthys lethostigma*	USA		HM035020.1	de Buron et al. [14]
*Philometra overstreeti*	W65	*Paralichthys lethostigma*	USA		HM035019.1	de Buron et al. [14]
*Philometra overstreeti*	W14	*Paralichthys lethostigma*	USA		HM035014.1	de Buron et al. [14]
*Philometra overstreeti*	W13	*Paralichthys lethostigma*	USA		HM035013.1	de Buron et al. [14]
*Philometra overstreeti*	W37	*Paralichthys lethostigma*	USA		HM035012.1	de Buron et al. [14]
*Philometra lagocephali*	D16-1	*Lagocephalus lunaris*	China	KP122959.1		Wang et al. [13]
*Philometra lagocephali*	D87	*Lagocephalus sceleratus*	China		KP122958.1	Wang et al. [13]
*Philometra lagocephali*	D18	*Lagocephalus sceleratus*	China		KP122957.1	Wang et al. [13]
*Philometra lagocephali*	D16-2	*Lagocephalus sceleratus*	China		KP122956.1	Wang et al. [13]
*Philometra lagocephali*	D12	*Lagocephalus lunaris*	China		KP122954.1	Wang et al. [13]
*Philometra lagocephali*	D8	*Lagocephalus sceleratus*	China		KP122953.1	Wang et al. [13]
*Philometra lagocephali*	D3	*Lagocephalus lunaris*	China		KP122952.1	Wang et al. [13]
*Philometra lagocephali*	D1	*Lagocephalus lunaris*	China		KP122951.1	Wang et al. [13]
*Philometra philippinensis*	KMAQ-2013—3	*Sphyraena forsteri*	Philippine	KC342905.1		Quiazon and Yoshinaga [28]
*Philometra philippinensis*	KMAQ-2013—2	*Sphyraena forsteri*	Philippine	KC342904.1		Quiazon and Yoshinaga [28]
*Philometra philippinensis*	KMAQ-2013—1	*Sphyraena forsteri*	Philippine	KC342903.1		Quiazon and Yoshinaga [28]
*Philometra lateolabracis*	TR115EK	*Epinephelus costae*	Turkey	JX456388.1		Keskin and Genc, unpubl.
*Philometra* sp.	1385	*Sphyraena novaehollandiae*	Australia	MZ274369.1		Barton et al. [29]
*Philometra* sp.	1386	*Sphyraena novaehollandiae*	Australia	MZ274368.1		Barton et al. [29]
*Philometra* sp.	1384	*Sphyraena novaehollandiae*	Australia	MZ274367.1		Barton et al. [29]
*Philometra* sp.	1383	*Sphyraena novaehollandiae*	Australia	MZ274366.1		Barton et al. [29]
*Philometra* sp.	13_1	*Lutjanus johnii*	Australia	MZ274364.1		Barton et al. [29]
*Philometra* sp.	12_1	*Lutjanus johnii*	Australia	MZ274363.1		Barton et al. [29]
*Philometra gracilis*	11_1	*Lutjanus johnii*	Australia	MZ274362.1		Barton et al. [29]
*Philometra* sp.	33_1	*Saurida tumbil*	Australia	MZ274357.1		Barton et al. [29]
*Philometra longa*	124_SS	*Belone belone*	Australia	MZ274356.1		Barton et al. [29]
*Philometra globiceps*	39_1	*Uranoscopus scaber*	Australia	MZ274354.1		Barton et al. [29]
*Philometra rara*	41_1	*Hyporthodus haifensis*	Australia	MZ274353.1		Barton et al. [29]
*Philometra arafurensis*	6_1	*Lutjanus sebae*	Australia	MZ274352.1		Barton et al. [29]
*Philometra iraqiensis*	30_1	Mugilidae	Australia	MZ274349.1		Barton et al. [29]
*Philometra diplectri*	haplotype3	Belonidae	Tunisia		MW326921.1	Moravec et al. [18]
*Philometra diplectri*	haplotype2	Belonidae	Tunisia		MW326920.1	Moravec et al. [18]
*Philometra nemipteri*		*Nemipterus virgatus*	Japan	FJ161975.1		Quiazon et al. [12]
*Philometra madai*		*Pagrus major*	Japan	FJ161974.1		Quiazon et al. [12]
*Philometra sciaenae*		*Pennahia argentata*	Japan	FJ161971.1		Quiazon et al. [12]
*Philometra* sp.		*Scomberomorus niphonius*	Japan	FJ161973.1		Quiazon et al. [12]
*Philometra carolinensis (outgroup)*	*S7*	*Menticirrhus americanus*	USA		JF894233.1	Palesse et al. [15]
*Dracunculus insignis (outgroup)*		*Procyon lotor*	USA	AY947719.1		Bimi et al. [30]

## Data Availability

The data presented in this study are available on request from the corresponding authors.
